# Letter regarding “Chronic mechanical irritation and oral squamous cell carcinoma: A systematic review and meta-analysis”

**DOI:** 10.17305/bjbms.2021.5939

**Published:** 2021-12

**Authors:** Jian Xie, Lang Li

**Affiliations:** Department of Cardiology, the First Affiliated Hospital of Guangxi Medical University, Guangxi Cardiovascular Institute, Nanning, Guangxi, China

Dear Editor:

We have read with a great interest the article by Gupta et al. [1] who performed a meta-analysis exploring the association between chronic mechanical irritation and oral squamous cell carcinoma. The conclusion of the meta-analysis is that chronic oral mucosa irritation has a significant association with oral squamous cell carcinoma, and the nature of association could be that of a potential cofactor (dependent risk factor) rather than an independent risk factor.

First, there could be a biased result from the repeated inclusion of studies, which were in the same study population, thus, duplicate populations in duplicate studies should be excluded from the research. Nonetheless, we have found that there were two studies in Table 1, the references 29 and 30, that might be the repeated articles included and affected the reliability of the results significantly. The studies in the references 29 and 30 were conducted by a group of authors in a hospital during a superposition researching time [2,3]. We suspect that they are two duplicate studies and therefore should be excluded from the study.

Second, as we all know, diverse carcinoma would have a diverse vulnerable population [4]. People belonging to different age groups, gender or ethnic groups and even to different cultures or particular lifestyles may have varying degrees of tumor susceptibility, which we always call individual differences [5]. Therefore, we consider that the basic characteristics of the included population should be listed in a table and analyzed statistically in order to determine whether there is a statistical difference between the exposed group and control group or not. The influence of the main risk factors for oral squamous cell carcinoma such as a history of smoking, drinking, and eating betel quid should be eliminated through statistical analysis. It is also necessary that these factors are listed in a separate table.

Third, the articles included in the research were all case-control and cross-sectional studies and five studies analyzed quantitatively have considered confounding factors. A propensity score matching could be added to eliminate the bias and confounding variables in included observational studies [6,7]. Generally speaking, each sample has multiple attributes, which means that many attributes should be considered when matching. Hence, adjusting the observational study data may lead to a more reliable conclusion.
